# Do Aspirin and Other Antiplatelet Drugs Reduce the Mortality in Critically Ill Patients?

**DOI:** 10.1155/2012/720254

**Published:** 2011-11-09

**Authors:** Wolfgang Lösche, Janina Boettel, Björn Kabisch, Johannes Winning, Ralf A. Claus, Michael Bauer

**Affiliations:** ^1^Center for Sepsis Control and Care, Jena University Hospital, Erlanger Allee 101, 07740 Jena, Germany; ^2^Department of Anesthesiology and Intensive Care Medicine, Center for Sepsis Control and Care, University Hospital Jena, Bachstraße 18, Gebäude 12, Eingang A, 07743 Jena, Germany

## Abstract

Platelet activation has been implicated in microvascular thrombosis and organ failure in critically ill patients. In the first part the present paper summarises important data on the role of platelets in systemic inflammation and sepsis as well as on the beneficial effects of antiplatelet drugs in animal models of sepsis. In the second part the data of retrospective and prospective observational clinical studies on the effect of aspirin and other antiplatelet drugs in critically ill patients are reviewed. All of these studies have shown that aspirin and other antiplatelet drugs may reduce organ failure and mortality in these patients, even in case of high bleeding risk. From the data reviewed here interventional prospective trials are needed to test whether aspirin and other antiplatelet drugs might offer a novel therapeutic option to prevent organ failure in critically ill patients.

## 1. Platelets in Systemic Inflammation and Sepsis

Sepsis and multiple organ failure are leading causes of death in critically ill patients. There is good evidence that blood platelets play an important role in the development of multiple organ failure (MOF) in these patients [1–3]. A decrease in the number of circulating platelets is very often observed when patients develop sepsis and MOF, and thrombocytopenia is a powerful predictor of mortality [4–6]. During systemic inflammation and infection platelets become activated as indicated by an increase in the number of CD62P-positive platelets and platelet-leukocyte conjugates [7–9]. Different mechanisms may contribute to platelet activation, including imbalance between plasma level of high molecular weight von-Willebrand factor and its cleaving protease, ADAMTS-13 [10–13], and binding of endotoxins to specific receptors at the platelet surface [14–16]. Adhesion of activated platelets within the microcirculation and formation of platelet aggregates contributes to vascular hyperpermeability as well as hypoperfusion [17–20]. 

However, platelets do not only contribute to the sepsis-associated disturbances of haemostasis, but they also significantly influence inflammatory processes:

- release of compounds with well-known pro- or anti-inflammatory effects such as cytokines, chemokines, and lipid mediators [21–26],
- activation of the complement system [27, 28],
- release of antibacterial compounds and, together with neutrophils, trapping of bacteria [25, 29–31],
- receptor-mediated adhesion to monocytes, neutrophils, and endothelial cells resulting in changes of cellular functions such as production of cytokines, chemokines, and reactive oxygen species as well as recruitment and immigration of leukocytes at the site of tissue damage [22, 25, 26, 32].

In summary, platelets may contribute to the development of MOF by disturbing blood flow as well as by modulating the systemic inflammation. Thus the question arises whether antiplatelet drugs may have a benefit on the outcome in critically ill patients, that is, in patients with systemic inflammation, severe infections, or sepsis.

## 2. Antiplatelet Drugs

Antiplatelet drugs are widely used in patients with cardiovascular disease for the secondary prevention of atherothrombotic events [34–36]. The mostly used drug is aspirin which is an irreversible inhibitor of cyclooxygenase. In platelets aspirin inhibits the formation of thromboxane A_2_ which is a potent platelet activator [37, 38]. Since aspirin also affects the cyclooxygenase in gastric mucosa which can lead to serious bleeding, it is used as an inhibitor of platelet function for the prevention in patients with risk for atherothrombosis in rather low dosage, that is, ≤325 mg/day, and in many patients at dosage lower than 160 mg/day [35, 36, 39–42].

Clopidogrel and the more recently developed drugs prasugrel and ticagrelor are rather specific inhibitors of platelet function. These drugs or their metabolic products interact with the platelet ADP receptor P2Y12, and they are used in combination or instead of aspirin [43–46]. Another group of antiplatelet agents are inhibitors of the glycoprotein IIb/IIIa complex on the platelet surface. These agents block the binding of fibrinogen to the receptor which is essential for platelet aggregation [47–49].

## 3. Anti-Inflammatory Effects of Antiplatelet Drugs in Patients with Cardiovascular Diseases

Many studies have shown that aspirin and clopidogrel not only reduce the risk of atherothrombotic events, but also reduce markers of systemic inflammation including C-reactive protein, soluble CD62P and CD54, pro-inflammatory cytokines, and platelet-leukocyte conjugates in these patients [50–54]. It is assumed that the anti-inflammatory effects of antiplatelet drugs are mediated by an inhibition of platelet activation [53]. 

## 4. Animal Studies on the Action of Antiplatelet Drugs in Systemic Inflammation and Sepsis

In the late seventies and in the eighties of the past century some studies on the beneficial effect of inhibitors of prostaglandin and thromboxane synthesis, including aspirin, on the survival in animal models of sepsis were reported [55–57]. It was shown that aspirin reduced platelet accumulation in the lung in a mouse model of endotoxinaemia [58, 59]. Other studies investigated the effects of glycoprotein IIb/IIIa inhibitors. Using monoclonal antibodies, a reduction of thrombotic microangiopathy and ischemic tissue injury in various animal models of endotoxinaemia or sepsis could be shown [60–62]. More recently, the effects of the ADP receptor antagonist clopidogrel in endotoxinaemia were tested. Evangelista et al. [63, 64] reported an inhibition of platelet-dependent leukocyte activation as well as an inhibition of the production of proinflammatory cytokines in mice after endotoxin administration. Our group could recently show that in a similar mouse model clopidogrel prevented endotoxin-induced thrombocytopenia, reduced fibrin deposition in lung tissue, and inhibited the upregulation of some genes, known to be involved in inflammation, in peripheral blood cells [65]. In a mouse model of polymicrobial sepsis Seidel et al. [66] demonstrated that clopidogrel reduced cell damage and liver dysfunction as indicated by reducing the sepsis-mediated increase in serum lactate dehydrogenase activity and serum bilirubin concentration. Using a rat model of endotoxin-induced systemic inflammation Hagiwara et al. [67] reported that clopidogrel attenuated the increase in serum levels of pro-inflammatory cytokines (TNF*α*, IL-6 and HMBG1) and the tissue injury in liver and lung. A benefit of clopidogrel was also shown in a rat model of chronic kidney disease [68].

## 5. Benefit of Aspirin and Other Antiplatelet Drugs in Critically Ill Patients

Based on the evidence that platelets play an important role in the development of organ failure in critically ill patients we performed three retrospective clinical studies. As critically ill patients are often elderly people we assumed that some of them might be on antiplatelet drugs for the prevention of thromboembolic events due to cardiovascular diseases. Indeed, 20–25% of the patients who were included in the analysis had a preexisting medication with aspirin or/and clopidogrel [33, 65]. Even if the administration of aspirin and/or clopidogrel was discontinued during the stay in hospital, inhibition of platelet function should persist for about one week [69, 70].

### 5.1. Patients Admitted to Hospital with Community Acquired Pneumonia

In a first study we analysed data from patients who were admitted to the hospital for community acquired pneumonia (CAP). Since statins are discussed to improve the outcome in critically ill patients [71–76], patients with prehospital use of statins were excluded from the study. Two hundred twenty-four patients were enrolled and 38 of them had a preexisting medication with aspirin and 8 were on clopidogrel or ticlopidin for at least 6 month prior to admission to hospital [65]. As endpoints of the study we used the length of stay in the hospital, and the admission to the intensive care unit (ICU) as an indicator of organ failure. Despite the fact that patients on antiplatelet drugs were about 12 years older when compared to those without such preexisting medication, they were less frequently admitted to ICU (9.1% versus 26.3%). This difference was more pronounced when age-matched subgroups were compared: 24.4% of patients without and only 5.0% of patients with antiplatelet drugs required ICU treatment. In the age-matched subgroups we observed also a significant shorter stay in hospital for the patients on antiplatelet drugs (13.9 ± 6.2 versus 18.2 ± 10.2 days). The beneficial effect of the preexisting medication with antiplatelet drugs was also obvious when stepwise logistic regression analysis was used to calculate the odds ratio for the need of ICU treatment. The following variables were included as independent variables: SOFA (sepsis-related organ failure assessment) score, plasma level of C-reactive protein, platelet and leukocyte counts (all measured at day of admission), age, gender, and the preexisting medication with antiplatelet drugs. Odds ratio of 0.32 (95% confidence interval: 0.10–1.00) for all patients and 0.19 (0.04–0.87) for the age-matched subgroup were obtained, indicating a marked reduction in organ failure by antiplatelet drugs [65].

### 5.2. Patients Admitted to an Intensive Care Unit

In a second study we analysed the data from 615 patients who were admitted to the intensive care unit (ICU) within 24 hours after arrival in hospital. From these patients 21% had a preexisting medication with aspirin (≤160 mg/d), and 4% were on clopidogrel or a combination of aspirin and clopidogrel. Patients on statins were excluded [33]. Patients were allocated to internal medicine as well as various surgery departments (general surgery, trauma surgery, neurosurgery, and gynaecology). Patients with and without antiplatelet drugs did not only differ in age (median: 72 versus 56 years), but also in the severity of their illness as measured by the APACHE (Acute Physiology and Chronic Health Evaluation) II score at the day of ICU admission (25 versus 19). Despite these marked differences in age and APACHE II score which are established risk factors of MOF, there was no difference in ICU mortality as clinical end point of the study. Stepwise logistic regression with APACHE II score, age, gender, and use of antiplatelet drugs indicated that in addition to age and APACHE II score the use of antiplatelet drugs had a highly significant impact on mortality. The calculated odds ratios amounted for age 1.04 (1.03–1.06), for APACHE II 1.16 (1.12–1.19), and for antiplatelet drugs 0.19 (0.12–0.33). That means that the prehospital use of antiplatelet drugs would reduce mortality by a factor of about 5 [33].

**Figure 1:**
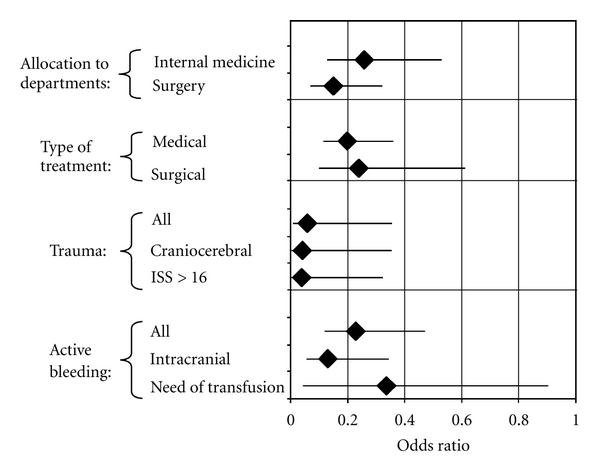
The figure summarises the effect of antiplatelet drugs (aspirin or/and clopidogrel) in patients admitted to an ICU as reported by Winning et al. [33]. Odds ratios for ICU mortality with 95% confidence intervals were calculated by stepwise logistic regression with age, gender, APACHE II score, and preexisting medication with antiplatelet drugs as independent variables.

Figure 1 illustrates the effects of antiplatelet drugs on subgroups of patients [33]. Surprisingly, in patients who were allocated to surgical departments, the preexisting medication with antiplatelet drugs was associated with a slightly better outcome when compared to those patients allocated to internal medicine department. In neurosurgery patients (46 with and 196 without antiplatelet drugs) an odds ratio for mortality of 0.12 (0.04–0.30) was calculated. In contrast, patients allocated to trauma surgery did not show any benefit from antiplatelet drugs (odds ratio = 0.92 (0.06–13.6)) [33]. However, in the subgroup of trauma patients (22 with and 159 without antiplatelet drugs) antiplatelet drugs seemed to exert an enormous benefit as an odds ratio as low as 0.06 (0.01–0.35) was calculated. This was true for patients with multiple trauma (injury severity score >16) as well as for patients with craniocerebral trauma (Figure 1). Even patients with active bleeding including those who needed transfusion or presented intracranial bleeding seemed to profit from the preexisting medication with antiplatelet drugs. Finally there was no marked difference in the beneficial effect of antiplatelet drugs between patients who received medical treatment and those with surgical treatment (Figure 1). Thus bleeding and/or high risk for bleeding seemed not to abolish or reverse the calculated benefit of antiplatelet drugs in the critically ill patients. However, one should consider the data obtained by the stepwise logistic regression analysis in some of the subgroups of patients with some caution as (i) the numbers of patients were sometimes rather low and (ii) patients without antiplatelet drugs were much younger (up to 30 years) when compared to those with such medication. Therefore we reevaluated the subgroups in a cohort of APACHE II score and age-matched patients using 2 × 2 table analysis [33]. Under this condition the beneficial effect of antiplatelet drugs in patients with active bleeding or a high bleeding risk was no longer significant, but the calculated odds ratios for mortality were in most of the subgroups in a range of 0.42 to 0.88. There were only two exceptions: in patients allocated to the trauma surgery department an odds ratio of 3.67 (0.38–42.2) was calculated. In contrast, a significant benefit on outcome was still obtained for neurosurgery patients (odds ratio = 0.32 (0.12–0.84)) [33].

For the present paper we have reevaluated our previously reported data [33] summarised above. We analysed the data of those patients who had a preexisting medication with only low-dose aspirin, and we excluded those patients who had clopidogrel or a combination of aspirin and clopidogrel. As for the entire group of patients with antiplatelet drugs we also found for the “only aspirin” subgroup large differences in age and APACHE II score when compared to patients without antiplatelet drugs. And again, there was no difference in mortality (Table 1). However using stepwise logistic regression analysis with mortality as dependent variable and age, APACHE II score, gender, and preexisting aspirin medication, we found that aspirin reduced the mortality by about 80% as indicated by an odds ratio of 0.20 (Table 2). Thus the calculated benefit of aspirin was in the same range as calculated for the entire group of patients with aspirin and/or clopidogrel as shown in Figure 1 and previously reported [33]. 

### 5.3. ICU Patients Presenting Severe Sepsis or Septic Shock

In a third, not yet published study, we analysed the clinical records of 834 patients who were admitted to ICU with severe sepsis or septic shock. About 20% of these patients received low-dose aspirin (Table 3). Exclusion criteria were the administration of other antiplatelet drugs (i.e., clopidogrel) or nonsteroidal anti-inflammatory drugs such as ibuprofen, diclofenac or indomethacin. As shown in Table 3, patients on aspirin were about 4.5 years older and presented a slightly higher APACHE II score at the day of ICU admission. Despite the differences in these both risk factors, ICU mortality was about one third lower in patients receiving aspirin when compared to those without such medication (Table 3).

When calculating the odds ratio for mortality by stepwise logistic regression with age, APACHE II score, and aspirin medication during ICU stay, we found a reduction in ICU mortality by aspirin of about 45% (Table 4).

### 5.4. Patients at Risk for Acute Lung Injury/Acute Respiratory Distress Syndrome

In June 2011 O'Neal et al. [73] published the data of a prospective study on the effects of the prehospital use of statins on the prevalence of severe sepsis and acute lung injury/acute respiratory distress syndrome (ALI/ARDS) in critically ill patients. The authors included 575 patients admitted to surgical or medical ICU. Exclusion criteria were admission to trauma or cardiovascular ICU, primary cardiac diagnoses, and age <40 years. From these patients 26% were on prehospital statins. Logistic regression analysis with age, gender, tobacco use, race, APACHE II score, statin use, and aspirin use indicated that patients on statin but not those on aspirin were less likely to have or to develop severe sepsis (odds ratio 0.62, 95% confidence interval 0.40–0.96) or ALI/ARDS (odds ratio 0.60, 95% confidence interval 0.36–0.99) during the first four days after ICU admission. Interestingly, the benefit of prehospital statins may be potentiated by prehospital aspirin. Patients with both prehospital statins and aspirin had the lowest rate of severe sepsis or ALI/ARDS when compared to those with statins alone or those without statins [73]. 

The association of prehospital aspirin therapy and ALI/ARDS was also investigated by Erlich et al. [77] and published in February 2011. The authors evaluated medical records of 161 patients with at least one major risk factor for ALI/ARDS but who did not meet criteria for ALI/ARDS at time of hospitalisation. Seventy-nine (49%) of the patients were on aspirin at hospital admission and 33 (21%) developed ALI/ARDS. Aspirin therapy was associated with a significantly lower rate of ALI/ARDS when compared to patients without aspirin (17.7% versus 28.0%; odds ratio 0.37, 95% confidence interval 0.16–0.84). The authors reported that the benefit of aspirin therapy remained significant after adjusting for various confounding variables [77]. A few months later the same group reported the results of a large multicenter international observational study on the association of prehospital aspirin therapy and ALI/ARDS [78]. Inclusion criteria were again at least on risk factor of ALI (aspiration, pneumonia, sepsis, shock, pancreatitis, high-risk trauma, or high risk surgery) and age >18 years. Patients with elective surgery were excluded. A total of 3855 patients were enrolled in the study. Twenty-five % of them were receiving aspirin at the time of hospitalisation and 6.2% developed ALI/ARDS. Patients with aspirin were significantly older (median and interquartile ranges: 70 (59–81) versus 51 (38–66) years) and had higher APACHE II scores (12 (8–16) versus 9 (5–14)). Univariate analysis indicated a reduced incidence of ALI/ARDS in patients with aspirin (odds ratio 0.65, 95% confidence interval 0.46–0.90). However this association was attenuated after adjusting for the propensity to receive aspirin therapy. An odds ratio (Cochran-Mantel-Haenszel pooled odds ratio) of 0.70 (0.48–1.03) was calculated in a stratified analysis based on deciles of the American Society of Anesthesiologists propensity scores [78]. 

## 6. Discussion

Animal studies and observational clinical studies reviewed here have provided some evidence that antiplatelet drugs may reduce organ failure and mortality in critically ill patients. In the clinical studies mostly or exclusively aspirin was used as the antiplatelet drug, whereas clopidogrel or GPIIb/IIIa inhibitors as rather specific antiplatelet drugs were predominantly used in animal models [60–68]. The benefit of clopidogrel and GPIIb/IIIa inhibitors in animal models and the benefit of low-dose aspirin in the observational clinical studies may indicate that the benefit of aspirin is indeed mediated by its effect on platelets. However, one cannot exclude the possibility that the benefit of antiplatelet drugs, including aspirin, is at least partially due to the underlying atherosclerotic vascular disease. It is well accepted that atherosclerosis is based on a chronic low-grade systemic inflammation as indicated by moderately increased levels of markers of inflammation, that is, cytokines, C-reactive protein, or fibrinogen [79–82]. It would be interesting to test the hypothesis that patients with chronic low-grade systemic inflammation have a decreased prevalence of severe sepsis and organ failure.

The use of antiplatelet drugs in critically ill patients seems not to be associated with unfavourable bleeding. A benefit of antiplatelet drugs was also evident in patients with an increased bleeding risk such as neurosurgery patients and not necessarily associated with high blood loss or worse neurological outcome [33]. This observation is in line with the recommendation of perioperative continuation of antiplatelet therapy in patients with high risk of cardio- and cerebrovascular events [83–86]. 

## 7. Conclusion

The data reviewed in the present paper may indicate that low-dose aspirin, as it is used in patients with cardiovascular, cerebrovascular, or peripheral vascular diseases, might offer a novel therapeutic option to prevent organ failure. This hypothesis warrants testing in prospective interventional trials. 

## Figures and Tables

**Table 1:** Age, gender, APACHE II score, and mortality in a subgroup of patients with and without an exclusive prehospital aspirin medication. The data were taken from Winning et al. [33].

	Control	ASA	Significance
Number	461	129	
Age (years)	52.2 ± 20.4	69.1 ± 9.3	*P* < 0.00001
Male/female (%)	56.8/43.2	57.4/42.6	n.s.
APACHE II	19.4 ± 8.5	26.1 ± 9.3	*P* < 0.00001
Mortality (%)*	38.4	38.8	n.s.

Data are given as mean ± standard deviation (sd), total numbers or %. Significances were calculated either by *t*-test for unpaired samples or by 2 × 2 table analysis. n.s. = not significant. * ICU mortality.

**Table 2:** Effect of aspirin on outcome of critically ill patients characterised in Table 1. Odds ratios for ICU mortality were calculated using data from our recently published study [33]. The model of stepwise logistic regression included age, gender, APACHE II score, and preexisting medication with aspirin as independent variables.

Variable	Odds ratio (95% CI)
Age	1.04 (1.03–1.06)
APACHE II score	1.16 (1.13–1.20)
Aspirin	0.20 (0.12–0.35)

**Table 3:** Age, APACHE II score, and mortality in patients admitted to ICU with severe sepsis or septic shock and with or without aspirin medication during ICU stay.

Variable	Aspirin during ICU stay		Significance
	No	Yes	
Number	647	187	
Age (years)	63.4 ± 14.0	67.9 ± 12.9	*P* < 0.0001
APACHE II	22.6 ± 9.2	24.1 ± 8.3	*P* < 0.05
Mortality (%)	33.8	23.5	*P* < 0.01

**Table 4:** Effect of ICU aspirin medication on outcome (odds ratio of mortality*) of patients with severe sepsis or septic shock. The model of stepwise logistic regression included age, APACHE II score and ICU medication with aspirin as independent variables. *ICU mortality.

Variable	Odds ratio (95% CI)
APACHE II score	1.05 (1.03–1.07)
Aspirin	0.55 (0.38–0.81)
